# *Acizzia errabunda* sp. nov. and *Ctenarytaina insularis* sp. nov.: Descriptions of two new species of psyllids (Hemiptera: Psylloidea) discovered on exotic host plants in New Zealand

**DOI:** 10.1371/journal.pone.0214220

**Published:** 2019-04-10

**Authors:** Francesco Martoni, Karen F. Armstrong

**Affiliations:** Bio-Protection Research Centre, Lincoln University, Lincoln, New Zealand; University of Saskatchewan College of Agriculture and Bioresources, CANADA

## Abstract

A recent molecular-based assessment of the psyllid fauna of New Zealand reported two genetically distinct, undescribed psyllid taxa on host plants not native to that country. Here, a morphological examination confirmed species-level variation that resulted in the description of two new psyllid species: *Acizzia errabunda* sp. nov. (Hemiptera: Psyllidae) from *Acacia baileyana* F. Muell and *Ctenarytaina insularis* sp. nov. (Hemiptera: Aphalaridae) from *Syzygium smithii* (Poir.) Nied. Furthermore, the examination of specimens from entomological collections and from observations recorded on an online database enabled a better understanding of the distribution and host plant associations of these psyllid species. The description of *A*. *errabunda* is based on material collected in both New Zealand and Australia from the same plant species, *A*. *baileyana*, whereas the psyllid *C*. *insularis* has been found to be present in Brunei and New Zealand on *S*. *smithii* and in New Caledonia on *Melaleuca quinquenervia* (Cav.) S. T. Blake.

## Introduction

The superfamily Psylloidea (Hemiptera: Sternorrhyncha) includes almost 4000 species described worldwide [1], with Australasia being a hotspot for the psyllid biodiversity [1, 2]. International interest in this group of hemipterans has increased since the discovery that some species are vectors of economically significant plant pathogens (e.g. [3–7]). This, coupled with climate change and globalization, has raised the risk of these species reaching areas and countries that would have not been inhabited a few decades ago [8–10]. As a consequence, psyllid taxonomy and systematics is of primary importance, not only for a better understanding of worldwide biodiversity, but also in order to recognise new or invasive species essential for international biosecurity and plant protection. Recent works on taxonomy and phylogenetics of psyllids have contributed towards this (e.g. [11–12]), including their placement within the order Hemiptera and their relationships with other hemipteroids [13].

Classification of the Psylloidea has been recently revised [11] and an increasing number of studies are using DNA sequence data to better understand the diversity and the phylogeny of taxa within it [12, 14–19]. Inevitably, new psyllid species are being described every year. For example, from the Hawaiian Islands 36 new species have been described in the genus *Pariaconus* [16] and seven in the genus *Swezeyana* [20], while 22 new species in the genera *Acizzia*, *Myotrioza* and *Trioza* have been described from Australia [14, 21].

The New Zealand psyllid fauna provides a good cross section of the worldwide psyllid biodiversity, including 24 genera across six of the eight families [22]. During the last 140 years, several taxonomists have described new species from this region (e.g. [23–26]). More recently, this group has come under close scrutiny in a biosecurity context due to the arrival there of the tomato/potato psyllid (TPP), *Bactericera cockerelli* Šulc. As vector of the plant pathogen ‘*Candidatus* Liberibacter solanacearum’, agent of the zebra chip disease, TPP has caused economically significant damage to New Zealand agriculture [7] and raised awareness of a need to know what species are present there [22].

A recent study of the New Zealand Psylloidea [18] used DNA barcoding [27] in support of morphological evidence and host plant associations to assess the biodiversity of this superfamily in the region. As a result, more than 20 new taxa were predicted to be new, undescribed species. Of these, two taxa were identified from host plants not native to New Zealand [18]. These were a *Ctenarytaina* sp. hosted by *Syzygium smithii* (Myrtaceae) a lilly pilly, but in New Zealand commonly known as ‘monkey apple’. Also an *Acizzia* sp. from *Acacia baileyana* (Fabaceae), the Cootamundra wattle. Both host plants are native to Australia [28], and therefore the origin of these psyllid species was initially considered to be from this country as well [18].

Worldwide, the genus *Ctenarytaina* (Aphalaridae) now includes 20 described species [1, 2] of which seven are present in New Zealand [22]. These are the endemic species *Ctenarytaina fuchsiae* (Maskell, 1890), *C*. *pollicaris* Ferris & Klyver 1932, and *C*. *clavata* Ferris & Klyver 1932, and the Australian native species *C*. *eucalypti* (Maskell, 1890), *C*. *longicauda* Taylor, 1987, *C*. *spatulata* Taylor, 1997 and *C*. *thysanura* Ferris & Klyver, 1932. Of all the *Ctenarytaina* psyllids worldwide, only one other species is associated with the plant genus *Syzygium*, being the recently described species *C*. *fomenae* from Western Cameroon [29]; there is no other *Ctenarytaina* associated with *Melaleuca*. Amongst other psyllid families, the species *Trioza adventicia* Tuthill, 1952 and *Trioza eugeniae* Froggatt, 1901 (both Triozidae) are the only other psyllids associated with *Syzygium smithii* [1, 22], while *Trioza jambolanae* Crawford, 1917 is associated with other *Syzygium* spp. in Bangladesh, China, India and West Himalaya [30, 31]. Similarly, the Australian psyllid *Boreioglycaspis melaleucae* (Moore, 1964) is the only other Aphalaridae sharing *Melaleuca* as the same host plant genus with *C*. *insularis*. In general, however, the association between psyllids of the genus *Ctenarytaina* and host plants of the family Myrtaceae is very well documented. For example, in Australia, many psyllid species in this genus are associated with *Eucalyptus*, such as *C*. *spatulata* Taylor, 1997, *C*. *eucalypti*, *C*. *bipartita* Burckhardt, Farnier, Queiroz, Taylor & Steinbauer, 2013, *C*. *gracilis* (Froggatt, 1901), *C*. *longicauda*, *C*. *obscura* (Froggatt, 1903) and *C*. *peregrina* Hodkinson, 2007 [2, 32].

The genus *Acizzia* (Psyllidae) includes 74 described species worldwide ([1]—excluding the *Psylla* taxa). The association between psyllids of the genus *Acizzia* and host plants of the family Fabaceae is strong, with at least 46 species documented as hosted by this legume family. Of these, 30 are on various plant species within the large *Acacia* genus [1]. Yen [33] suggested the number of Australian *Acizzia* spp. associated with *Acacia* may range between 58 and 77, however many of these species remain undescribed. New Zealand is home to 10 described species of *Acizzia* [22, 26]. These are *Acizzia acaciae* (Maskell, 1894), *A*. *acaciaebaileyanae* (Froggatt,1901), *A*. *albizziae* (Ferris & Klyver, 1932), *A*. *conspicua* Tuthill, 1952, *A*. *dodonaeae* Tuthill, 1952, *A*. *exquisita* Tuthill, 1952, *A*. *hakeae* Tuthill, 1952, *A*. *jucunda* Tuthill, 1952, *A*. *solanicola* Kent & Taylor, 1932 and *A*. *uncatoides* (Ferris & Klyver, 1932) [22, 34].

The species described here increase the number of taxa belonging to their respective genera in New Zealand. Therefore, the descriptions reported here are aimed to provide a tool for their identification while contributing to a better understanding of the New Zealand biodiversity.

## Materials and methods

### Materials collected

Psyllids collections were performed by beating plant leaves and branches on trays and by collecting the fallen insects using an entomological aspirator. After collection, all specimens were preserved in high grade ethanol and stored at -20°C.

One hundred six specimens of *Ctenarytaina insularis* sp. nov. were collected from two distinct locations in the urban area of Auckland and from one location in Wellington, New Zealand (Table 1). On the 21^st^ of February 2014 a first population was collected by S. Bulman (The New Zealand Institute of Plant and Food Research) from a *Syzygium smithii* in the urban area of Auckland, while a second population was collected by FM on the 23^rd^ of March 2015, in Fowlds Park, Auckland, also from *S*. *smithii*. A single psyllid was collected by FM on the 28^th^ of February 2016 on a *Pittosporum eugenioides*, in the urban area of Wellington, near Kelburn Park. A total of four specimens from these three locations, were previously used for DNA analysis [18] and partial sequences of the subunit 1 of the cytochrome oxidase gene (COI) are available on GenBank with accession numbers MG132452- MG132455. Additionally, samples from a fourth location (Auckland, New Zealand) previously collected by C.F. Hill (1997) on *S*. *smithii* and stored in high grade ethanol since that time, were provided by S. Bennett (New Zealand Ministry for Primary Industries, MPI) for examination (Table 1).

**Table 1:** Species distribution and collection sites. Country, location and host plant are reported for all the species examined in this study. This includes specimens collected by the authors (**SC**), loaned from museums and collections (**SL**) or provided by colleagues (**SP**), and observations reported on online database—only when including pictures that allowed identification (**O**). The star (*) highlights the only instance where a single insect was collected and, consequently, the host plant attribution is not confirmed.

Species	Country	Location	Host plants	Source
C. insularis sp. nov.	Brunei	Sungai Temburong.	*Syzygium* sp.	SL
	New Zealand	Auckland, Mt. Albert.	*Syzygium smithii*	SP/SL
	New Zealand	Auckland, CBD.	*Syzygium smithii*	SC
	New Zealand	Auckland, Fowlds Park.	*Syzygium smithii*	SC
	New Zealand	Auckland, Henderson Park.	*Syzygium smithii*	O
	New Zealand	Auckland, St. John.	*Syzygium smithii*	O
	New Zealand	Wellington, Kelburn Park.	Pittosporum eugenioides*	SC
	New Caledonia	Hiénghene.	*Melaleuca quinquenervia*	SL
	New Caledonia	Tiebaghi mines.	*Melaleuca quinquenervia*	SL
	New Caledonia	Mount Toro.	*Melaleuca quinquenervia*	SL
	New Caledonia	Mount Koghis.	*Melaleuca quinquenervia*	SL
	New Caledonia	10 km South of Poum.	*Melaleuca quinquenervia*	SL
A. errabunda sp. nov.	New Zealand	Lincoln, Mahoe Reserve.	*Acacia baileyana*	SC
	New Zealand	Spring Grove.	*Acacia baileyana*	SP
	New Zealand	Auckland, Point England.	*Acacia baileyana*	O
	New Zealand	Auckland, Glendowie.	*Acacia baileyana*	O
	New Zealand	Auckland, Stonefields.	*Acacia baileyana*	O
	Australia	South Australia, Mt. Barker.	*Acacia baileyana*	SC

Eighty specimens of *Acizzia errabunda* sp. nov. were collected using the same procedure as above, from one location in South Australia (by FM and G. Taylor, 6^th^ of November 2014 near Mt Barker) and one in New Zealand (by FM, 17^th^ of July 2015 in the Mahoe Reserve, Lincoln, Canterbury), both from *Acacia baileyana* (Table 1). A total of six specimens, two from each of these populations, were previously used for DNA analysis [18] and partial sequences of COI are available on GenBank with accession numbers MG132221- MG132226. In addition to these, six individuals previously collected in Spring Grove, New Zealand, in December 2015 by H. Evans (following the same methods of [18]) were provided by C. Lange and S. Fowler (AgResearch Ltd) (Table 1). These specimens were preserved in high grade ethanol and had been stored at -20°C.

### Materials examined from established collections

Additionally, samples belonging to different species were examined for a better understanding of the morphological differences between these and the newly described species. Multiple specimens of *Acizzia acaciaebaileyanae* (Froggatt, 1901), *Acizzia jucunda* (Tuthill, 1952), *Ctenarytaina fuchsiae* (Maskell, 1890), *Ctenarytaina clavata* Ferris & Klyver 1932 and *Ctenarytaina pollicaris* Ferris and Klyver 1932 from the Lincoln University Entomology Collection (**LUNZ**) were morphologically examined using the keys as detailed below. Furthermore, the holotype of *Acizzia jucunda* was provided by the New Zealand Arthropod Collection (**NZAC**), while the holotypes of *Ctenarytaina distincta* and *C*. *lulla* were obtained from the Bernice P. Bishop Museum of Honolulu, Hawaii (**BPBM**).

Unidentified specimens of *Ctenarytaina* spp. from New Zealand, New Caledonia and Brunei (details given below) were provided by the British Museum of Natural History (**BMNH**) in London, UK, together with specimens of *Acizzia jucunda* from Australia.

Preparation of specimens followed the work of Taylor and colleagues [14]. Morphology of adult characters followed Hodkinson and White [35] and that of immature characters followed White and Hodkinson [36]. Photographs were taken using a Nikon DS‐Ri2 camera connected to a Nikon SMZ25 microscope. Pictures were the result of stacking images using the software Nikon NIS‐Elements D v4.5. The magnification of each picture depended upon the dimension of the insects and of the morphological character of interest. Plates were prepared using GIMP version 2.8.14. Drawings were made using Inkscape v.0.92.3. All of the psyllid specimens collected, the holotypes and part of the paratypes have been deposited at the LUNZ. The remaining paratypes have been deposited at the BMNH.

Label data of holotypes are reported here using the conventions of Brown [37]: each label is delimited using quotes (‘…’), while lines are indicated with a solidus (/) and metadata are reported in curly brackets ({…}).

### Nomenclatural acts

The electronic edition of this article conforms to the requirements of the amended International Code of Zoological Nomenclature (ICZN) [38, 39], and hence the new names contained herein are available under that Code from the electronic edition of this article. This published work and the nomenclatural acts it contains have been registered in ZooBank, the online registration system for the ICZN. The ZooBank LSIDs (Life Science Identifiers) can be resolved and the associated information viewed through any standard web browser by appending the LSID to the prefix “http://zoobank.org/”. The LSID for this publication is: urn:lsid:zoobank.org:pub:434FBA6F-A948-4005-824B-D5FD495BE6EC.

The electronic edition of this work was published in a journal with an ISSN and has been archived and is available from the following digital repositories: PubMed Central and LOCKSS.

## Results

The specimens that were examined for this work, including museum specimens, and concluded to belong to the new species *A*. *errabunda* and *C*. *insularis* are reported in Table 1.

### Taxonomy

#### *Ctenarytaina insularis* Martoni & Armstrong, 2019

LSID: urn:lsid:zoobank.org:act:4419FC71-631B-42C9-84FF-FFDA7B91AEF7

(Figs 1, 2 and 3)

**Fig 1:**
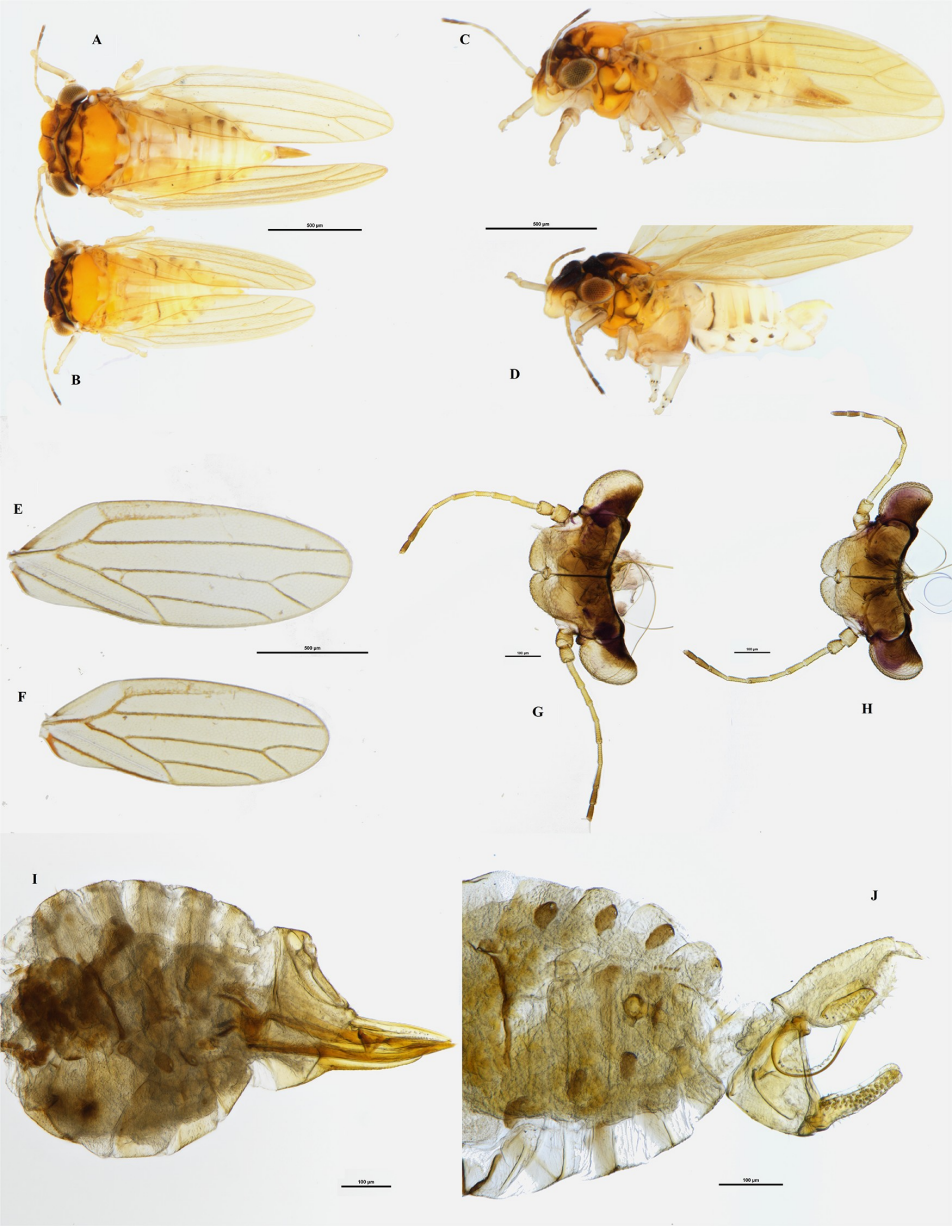
*Ctenarytaina insularis* sp. nov. Habitus dorsal view of female **(A)** and male **(B)**; habitus lateral view of female **(C)** and male **(D)**; wings of female **(E)** and male **(F)**; head dorsal view of female **(G)** and male **(H)**; lateral view of terminalia of female **(I)** and male **(J)**. Scale bar length = 500μm **(A-F)** and 100μm **(G-J)**.

**Fig 2:**
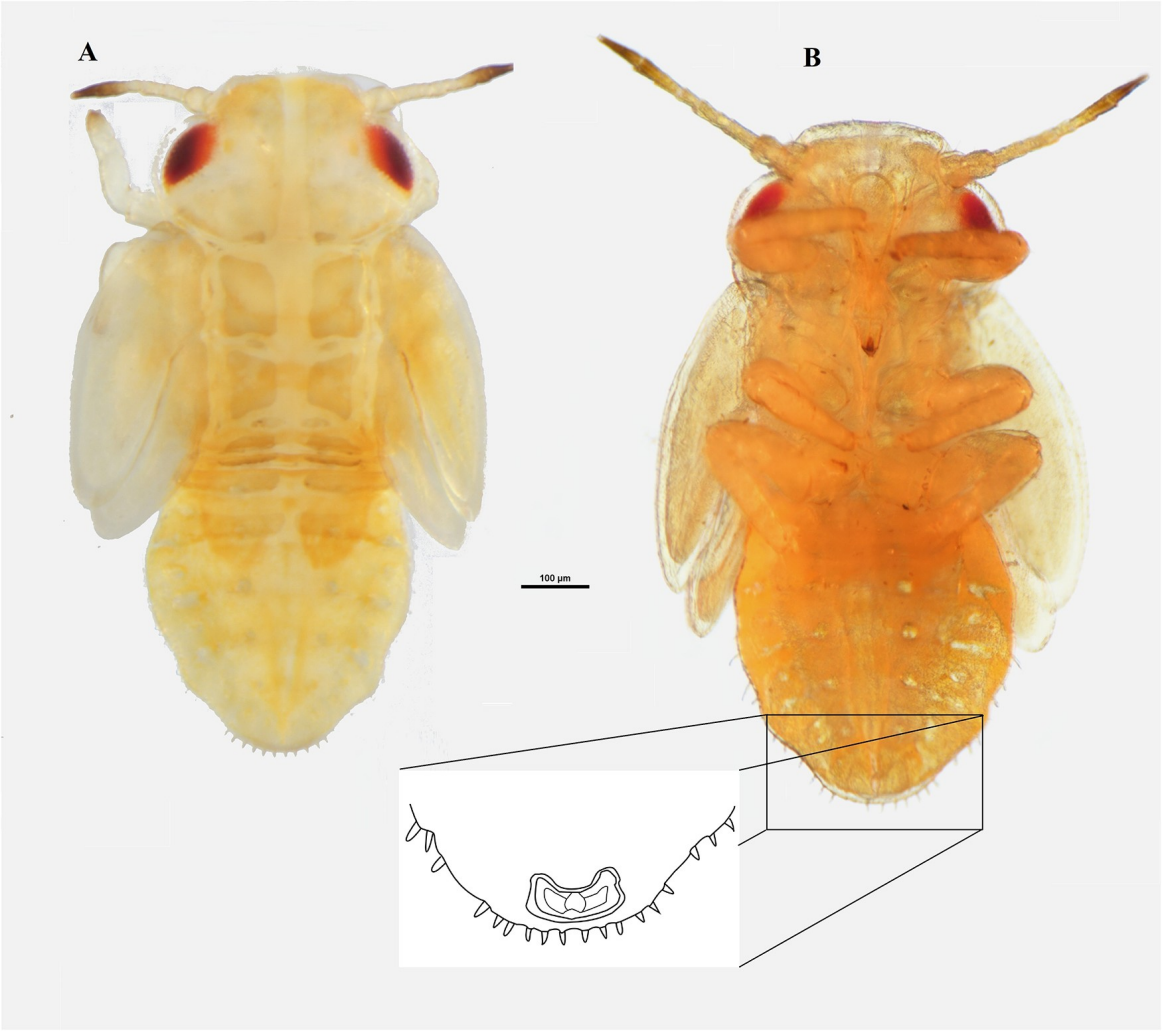
Nymph of *Ctenarytaina insularis* sp. nov. Habitus dorsal view **(A)** and habitus ventral view **(B)** with particular emphasis on the anal opening. Scale bar = 100μm.

**Fig 3:**
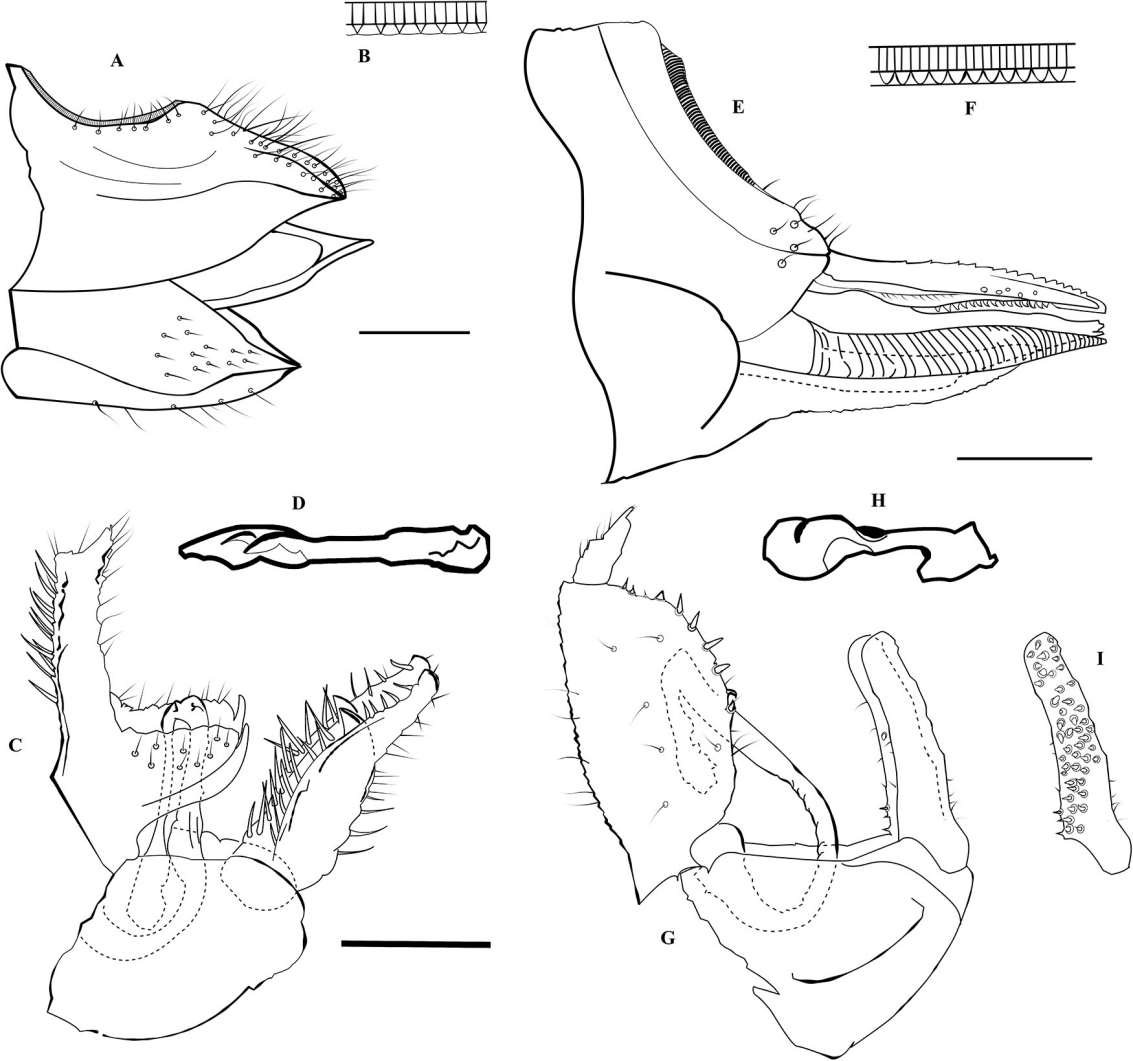
**Details of terminalia** for ***A*. *errabunda***, female terminalia **(A)**, anal ring **(B)** and male terminalia **(C)** with aedeaegus **(D)**, and ***C*. *insularis***, female terminalia **(E)**, anal ring **(F)** and male terminalia **(G)** with aedeaegus **(H)** and inner side of paramere **(I)**.

### Materials examined

Holotype: **♂** mounted on card triangle, deposited at the LUNZ. Labels: ‘NEW ZEALAND, AK / Auckland, Fowlds Park / 23 Mar 2015 F. Martoni / On *Syzygium smithii*.’ {Printed on white card}. ‘ID 127—Martoni F. 2017 / PhD Thesis. Lincoln University, / Canterbury–New Zealand.’ {Printed on white card}. ‘HOLOTYPE ♂ / *Ctenarytaina insularis /* Martoni & Armstrong 2019’ {Printed on red card}.

Paratypes: **3 ♀♀** mounted on card triangle, deposited at the LUNZ. Labels: ‘NEW ZEALAND, AK / Auckland, Fowlds Park / 23 Mar 2015 F. Martoni / On *Syzygium smithii*.’ {Printed on white card}, ‘ID 127—Martoni F. 2017 / PhD Thesis. Lincoln University, / Canterbury–New Zealand.’ {Printed on white card}, ‘PARATYPE ♀ / *Ctenarytaina insularis /* Martoni & Armstrong 2019’ {Printed on blue card}; **1 ♂, 1 ♀** mounted on microscope slide, deposited at the LUNZ. Labels: ‘PARATYPE / *Ctenarytaina insularis* / Martoni & Armstrong / 2019’ {Hand-written on white card}, ‘NEW ZEALAND, AK / Auckland—Mt Albert / 4 Nov 1997—C. F. Hill / on *Syzygium smithii*.’ {Hand-written on white card}; **2 ♂♂, 1 ♀** mounted on microscope slide, deposited at the LUNZ. Labels: ‘PARATYPE / *Ctenarytaina insularis* / Martoni & Armstrong / 2019’ {Hand-written on white card}, ‘NEW ZEALAND, AK / Auckland, Fowlds Park / 23 Mar 2015 F. Martoni / On *Syzygium smithii*.’ {Hand-written on white card}; **8 ♂♂, 5 ♀♀** mounted on 3 pins, deposited at the BMNH. Labels:

‘PARATYPE/ *Ctenarytaina insularis* / Martoni & Armstrong / 2019’ {Hand-written on white card}, ‘N. ZEALAND: / Auckland / Mt Albert / 26 VI 1997 / K. Froude leg P.Dale.’ [Hand-written on white card], ‘*Acmena smithii*.’ {Hand-written on white card}.

### Diagnosis

*Ctenarytaina insularis* can be identified by the following combination of characters: small size, dark-brown colouration of the head and lighter (orange-yellowish) colouration to the rest of the body, rectangular vertex, and antennae as long as width of head. Female genitalia are shorter than the rest of abdomen, but of elongated shape and characterised by a pronounced post-anal bump and by both subgenital and proctiger tips pointing inward. The subgenital plate is shorter than the proctiger, which is straight and not downcurved. Male terminalia are pointy, with apical part of the parameres square and lacking setae on its inner border.

**Fig 4:**
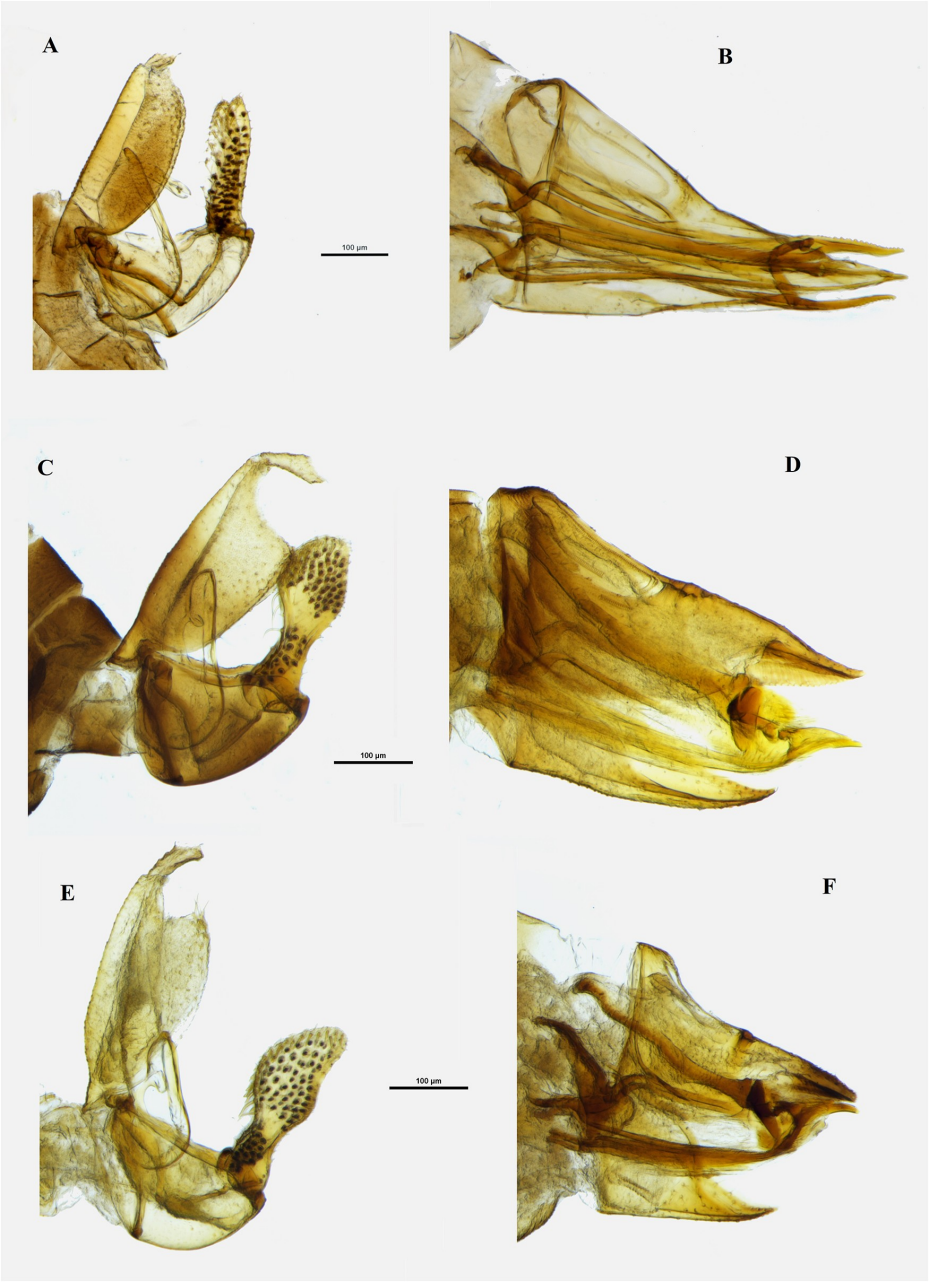
**Details of terminalia** for ***C*. *fuchsiae***, male terminalia **(A)**, and female terminalia **(B)**, ***C*. *pollicaris***, male terminalia **(C)**, and female terminalia **(D)**, and ***C*. *clavata***, male terminalia **(E)**, and female terminalia **(F)**. Scale bar length = 100μm.

When compared with the *Ctenarytaina* species inhabiting New Zealand, the colour and size of *C*. *insularis* are similar to those of *C*. *fuchsiae*. The female terminalia of *C*. *insularis* (Figs 1I, 3E and 3F) are elongated in a similar way to those of *C*. *fuchsiae* (Fig 4B). However, they can be clearly distinguished by the more pronounced post-anal bump, by the tip of both subgenital plate and proctiger pointing inward (and not outward as in *C*. *fuchsiae*), and by the fact that the subgenital plate is shorter than the proctiger (Figs 1I, 3E and 3F). Similarly, the shape of the male terminalia of *C*. *fuchsiae* (Fig 4A) are differentiated by the tips of the parameres being pointy (and not rounded as in *C*. *insularis*, Figs 1J and 3G–3I), the shape of the proctiger being less rounded, and the lack of pronounced setae on its inner border. Additionally, the plant host for *C*. *fuchsiae* is *Fuchsia excorticata* (Onagraceae), while that for *C*. *insularis* is *Syzygium smithii* (Myrtaceae, see below). No New Zealand native *Ctenarytaina* are hosted by *Syzygium*. Rather, two species, *C*. *pollicaris* Ferris & Klyver, 1932 and *C*. *clavata* Ferris & Klyver, 1932, live on *Kunzea* spp. and *Leptospermum* spp. (both Myrtaceae) respectively. Compared to these other *Ctenarytaina* species, the dark orange/brown/yellow colour of *C*. *insularis* differs greatly from *C*. *pollicaris*, which is completely dark/black and *C*. *clavata* which is more uniformly yellow [26]. Also, while the female terminalia of *C*. *insularis* are slightly similar to those of *C*. *pollicaris* (Fig 4D), they narrow distinctively to a thin end immediately after the circum-anal ring. In addition, both the clavate male parameres of *C*. *pollicaris* (Fig 4C) and *C*. *clavata* (Fig 4E) differ greatly from the thin parameres of *C*. *insularis*.

Outside New Zealand, the morphology of *C*. *insularis* appears closely related to those of *C*. *lulla* (Tuthill, 1942) and *C*. *distincta* (Tuthill, 1943), two species endemic to the Society Islands (French Polynesia). Despite these species being described solely based on female specimens [40, 41], the original description and the subsequent examination of the holotypes clearly identified these as different species. In fact, the female proctiger of *C*. *distincta* is down-curved (and not straight as in *C*. *insularis*; Figs 1I, 3E and 3F) and the subgenital plate is almost as long as the proctiger (while it is clearly shorter in *C*. *insularis*; Figs 1I, 3E and 3F). Additionally, upon inspection of the holotype, the colour of the body was darker, brown to dark brown, and the fore wing presented a darker band covering its proximal half (absent in *C*. *insularis*, Fig 1E and 1F). Similarly, *C*. *lulla* presents a vertex narrowed anteriorly (also shown in the original drawings [40]), antennae only ¾ of head’s width and female genitalia longer than the rest of abdomen; while *C*. *insularis* has rectangular vertex not narrowing anteriorly (Fig 1G and 1H), antennae as long as width of head (Fig 1G and 1H), and female genitalia shorter than rest of abdomen (Fig 1A, 1C and 1I). The holotype of *C*. *lulla* also showed a shorter vein M and longer veins M_1+2_ and M_3+4_, resulting in the cell m_1_ being thinner and longer than that of *C*. *insularis*.

When considering the host plant association, the only other *Syzygium*-inhabiting *Ctenarytaina* is the recently described *Ctenarytaina fomenae* Soufo *et al*. 2018 [29], recorded in the African region of Western Cameroon. Beside the geographical separation between *C*. *fomenae* and *C*. *insularis*, the two species can be distinguished morphologically by the shape of the apical part of the parameres in *C*. *insularis* being straight and not slightly back turned as for *C*. *fomenae*.

A total of 83 specimens of *C*. *insularis* were identified amongst the samples provided by the BMNH. Of these, nine slide-mounted specimens (into three microscope slides) and 13 pinned specimens were originally collected in New Zealand in 1997 by K. Froude and P. Dale, from *Syzygium smithii* (Table 1). Another 32 slide-mounted specimens (into 20 microscope slides) and 14 pinned samples were collected from five different areas of New Caledonia in 1982 by D. Hollis, from *Melaleuca quinquenervia* (Myrtaceae) (Table 1). Lastly, five adult females and 10 nymphs (on four microscope slides) were collected from *Syzygium* in Brunei, on the 31^st^ of October 1992 by J.H. Martin, and provided by the BMNH. These, despite not including male specimens, could be unambiguously identified as *C*. *insularis*.

### Colouration

**Adult**. Overall colour of the body dark orange/brown, with head and thorax dark brown or black while the abdomen remains of a lighter pale yellow (Fig 1A–1D). Eyes dark. Genal processes yellow. Antennae are light orange, with the last two segments dark brown. Wings are hyaline with darker veins (Fig 1E and 1F). Male terminalia are pale yellow. Female terminalia are darker, with an orange proctiger, similar to the colour of the thorax.

**Nymphs**. Habitus as in Fig 2. The cephalo-thoracic plate is light orange/yellow; eyes are red with a darker brownish spot laterally; antennae light orange/yellow, with the last two segments of a darker brown; thoracic sclerites just slightly darker than the rest of the body; forewing and hindwing pads almost white; caudal plate slightly glossy.

### Structure

**Adult**. Body short and broad. Head: vertex short and broad, 0.45 times as long as wide; genal processes short and rounded. Antennae quite short, as long as head if not shorter. Thorax short and broad. Fore wing 2.35 times as long as width of head and 2.7 times as long as wide. Vein Rs longer than M (up to 1.6 times), both veins are straight and parallel with vein Cu1a, until Cu1a abruptly deviates to the margin of the wing. Male terminalia quite slender, with proctiger longer than parameres. Parameres thin and slender internally covered in setae, except for a triangular area at the base. Female terminalia straight and moderately long with proctiger longer than subgenital plate (up to 1.4 times), wider in the proximal part and narrow to the point.

**Nymphs**: Habitus as in Fig 2. Distribution of capitate setae on lateral margin of caudal plate shows 12 setae for each side divided into two groups of three setae and a caudal group of six. However, the six setae in caudal position for each side are grouped together with those of the other side, resulting in a group of 12 setae in caudal position (Fig 2). Circum-anal pore ring in ventral position (Fig 2B).

### Measurements and ratios

Measurements are in mm (5 ♂, 5 ♀). Length of body (vertex to terminalia) ♂ 0.93–1.31, ♀ 1.01–1.44; length of body (vertex to apex of folded wings) ♂ 1.29–1.63, ♀ 1.55–1.92; width of head (HW) ♂ 0.55–0.58, ♀ 0.58–0.69; length of genal processes (GCL) ♂ 0.03–0.04, ♀ 0.04–0.05; length of vertex (VL) ♂ 0.14–0.16, ♀ 0.13–0.17; width of vertex (VW) ♂ 0.31–0.36, ♀ 0.35–0.37; length of antenna (AL) ♂ 0.53–0.57, ♀ 0.53–0.60; length of fore wing ♂ 1.25–1.33, ♀ 1.41–1.54; width of fore wing ♂ 0.45–0.51, ♀ 0.51–0.56; length of vein Rs ♂ 0.92–1.02, ♀ 1.08–1.18; length of vein M (M) ♂ 0.60–0.67, ♀ 0.67–0.76; length of vein M1+2 (M1) ♂ 0.24–0.31, ♀ 0.30–0.36; marginal width of cell m1 ♂ 0.12–0.17, ♀ 0.16–0.18; marginal width of cell cu1 ♂ 0.37–0.40, ♀ 0.41–0.49; length of vein Cu1b ♂ 0.09–0.14, ♀ 0.13–0.15; length (height) of proctiger (PL) ♂ 0.21–0.22; length of paramere ♂ 0.14–0.16; length of proximal aedeagal segment ♂ 0.29–0.31; length of distal aedeagal segment ♂ 0.06–0.09; length of proctiger (PL) ♀ 0.36–0.41; length of circum-anal ring (CL) ♀ 0.15–0.17; length of subgenital plate (SL) ♀ 0.29–0.34.

Ratios: GCL:VL ♂ 0.24–0.27, ♀ 0.24–0.31; VL:VW ♂ 0.40–0.49, ♀ 0.40–0.49; VL:HW ♂ 0.25–0.29, ♀ 0.22–0.30; AL:HW ♂ 0.97–1.01, ♀ 0.80–0.99; PL:HW ♂ 0.36–0.40, ♀ 0.53–0.70; PL:CL ♀ 2.40–2.52; PL:SL ♀ 1.08–1.40; WL:HW ♂ 2.31–2.41, ♀ 2.09–2.56; WL:WW ♂ 2.61–2.78, ♀ 2.68–2.84; Rs:M ♂ 1.45–1.61, ♀ 1.55–1.62; M1:M ♂ 0.37–0.49, ♀ 0.43–0.52.

### Etymology

The latin word “*insularis*” means “of the island”. This was chosen since this psyllid was collected in Brunei, New Caledonia and in New Zealand, making it an “insular” psyllid.

### Distribution

In New Zealand, *Ctenarytaina insularis* was collected for this study from four locations, all in the North Island (Table 1). Three of these were within the urban area of Auckland, including Mt. Albert and Fowlds Park, while the fourth was in Wellington. Additionally, populations from other areas of Auckland (Henderson Park and the suburb of St. John) were observed by S. Thorpe and pictures and information allowing identification were provided on the website iNaturalist (https://www.inaturalist.org). Other collections included 21 specimens from an urban area of Auckland (Mt. Albert), New Zealand, in 1997, by P. Dale and K. Froude on *Syzygium smithii* and 48 specimens from New Caledonia in 1982 by D. Hollis, on *Melaleuca quinquenervia* (Myrtaceae). Specifically, eight microscope slide-mounted samples were collected in the town of Hienghène, 26 samples (mounted on 12 slides) on Mt. Koghis, 12 pinned samples in the area of Noumea (Mt. Toro) and one sample each in Poum and near Tiebaghi mines (Table 1). All the specimens collected in Brunei were collected by J.H. Martin from the same location in the Temburong district, Sungai Temburong.

### Host plant

All the specimens from New Zealand were collected from *Syzygium smithii* (Myrtaceae), except for a single specimen collected from Lemonwood (*Pittosporum eugenioides*), in Wellington. Considering that both immature stages and adult individuals were recorded in high numbers from *S*. *smithii* on multiple occasions, this plant is reported here as *Ctenarytaina insularis*’ host plant. This is in accordance with the definition of host plant made by Burckhardt and colleagues [42], stating that all the life stages must be present on the plant for it to be defined as host plant. Similarly, all the specimens from Brunei were collected from a *Syzygium* sp. and they included immature stages. On the other hand, the specimens from New Caledonia were collected from *Melaleuca quinquenervia*. These also included both immature stages and adults. While it is not extremely common for a psyllid species to have host plants belonging to different genera [43], it is not rare to record different host plants within the same family [43]. Therefore, considering that both *M*. *quinquenervia* and *S*. *smithii* belong to the family Myrtaceae, it is plausible to assume that these are both host plant to *C*. *insularis* in the respective countries.

### Remarks

The first official report of *Ctenarytaina insularis* in New Zealand dates back to 2010 [34] as *Ctenarytaina* sp. on *Syzygium*. This was informed by field collections performed in the area of Auckland in 1997 (S. Bennett, personal communication), therefore this species is already included, as *Ctenarytaina* sp. within the 99 taxa reported in the most recent checklist of the New Zealand psyllids [22].

There are no reports for either *Ctenarytaina* spp. or Aphalaridae spp. in New Caledonia [1], therefore *C*. *insularis* would be the first member of this genus and family, there.

### Taxonomy

#### *Acizzia errabunda* Martoni and Armstrong, 2019

LSID: urn:lsid:zoobank.org:act:837B901D-F6A7-421D-AF39-345F610A79E5

(Figs 3, 5 and 6)

**Fig 5:**
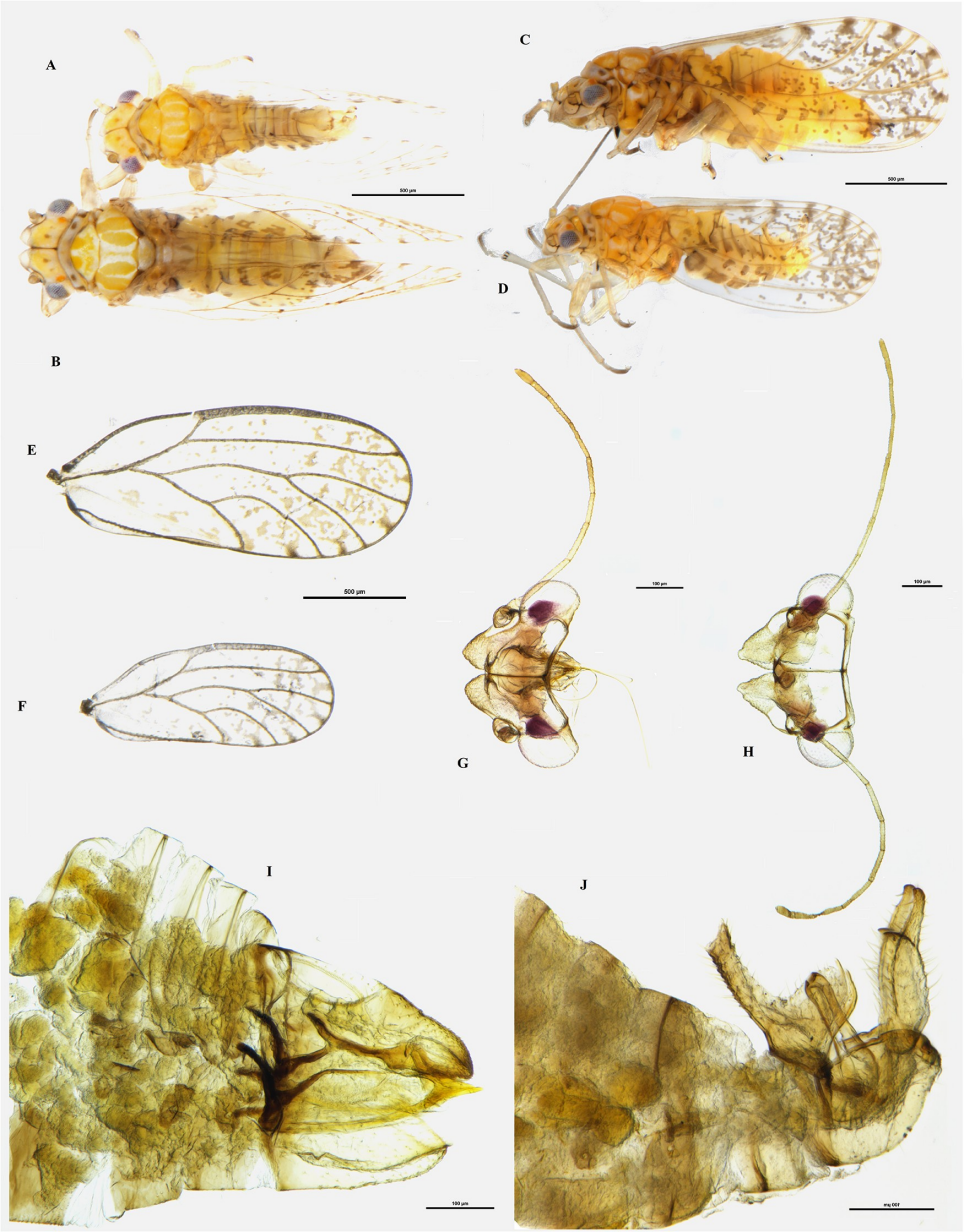
*Acizzia errabunda* sp. nov. Habitus dorsal view of male **(A)** and female **(B)**; habitus lateral view of female **(C)** and male **(D)**; wings of female **(E)** and male **(F)**; head dorsal view of male **(G)** and female **(H)**; lateral view of terminalia of female **(I)** and male **(J)**. Scale bar length = 500μm **(A-F)** and 100μm **(G-J)**.

**Fig 6:**
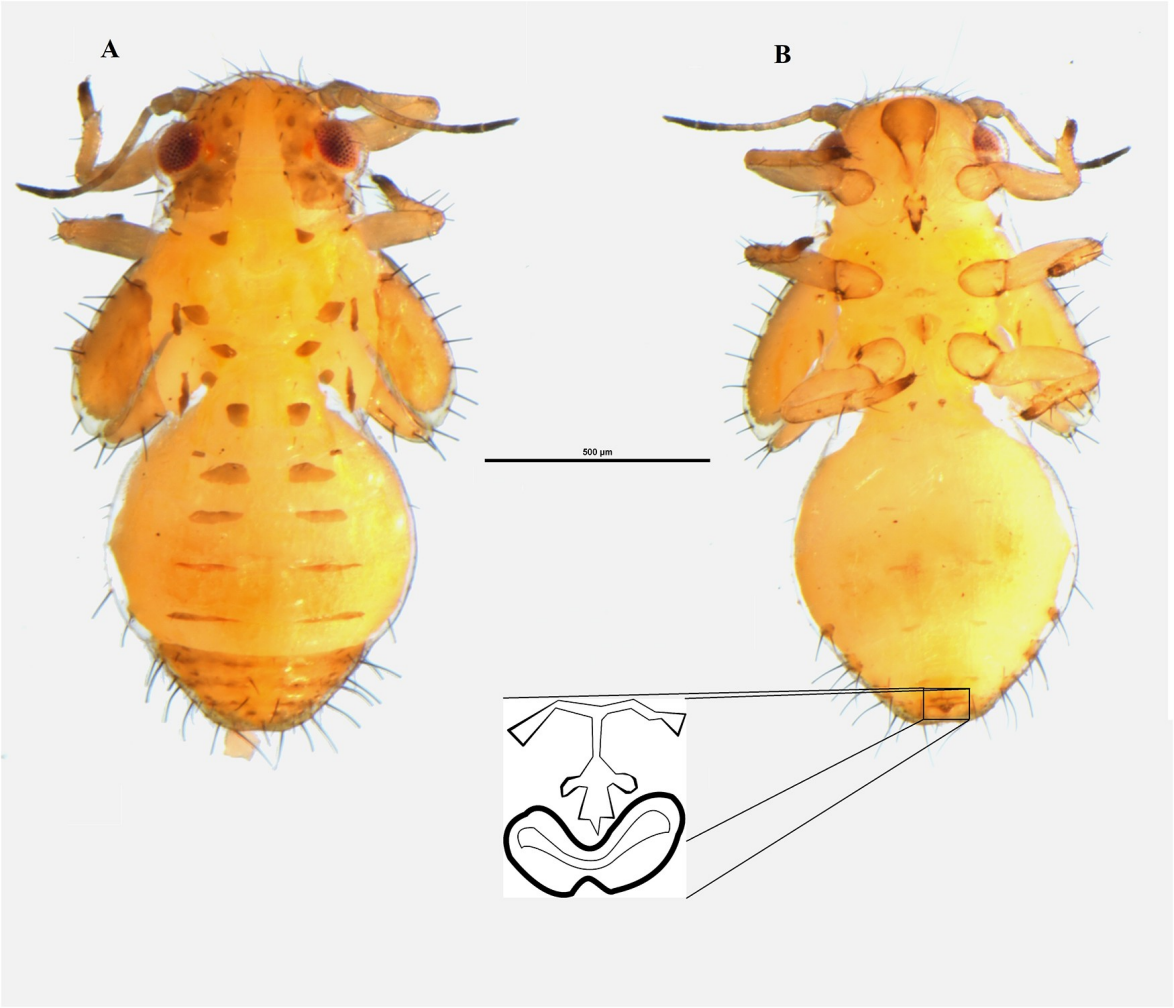
Nymph of *Acizzia errabunda* sp. nov. Habitus dorsal view **(A)** and habitus ventral view **(B)** with particular emphasis on the anal opening. Scale bar = 500μm.

### Materials examined

Holotype: **♂** mounted on card triangle deposited at the LUNZ. Labels: ‘NEW ZEALAND, MC / Lincoln, Mahoe Reserve / 17 Jul 2015 F.Martoni / on *Acacia baileyana’* {Printed on white card}, ‘ID 180—Martoni F. 2017 / PhD Thesis. Lincoln University, / Canterbury–New Zealand.’ {Printed on white card}, ‘HOLOTYPE ♂ / *Acizzia errabunda* / Martoni & Armstrong 2019’ {Printed on red card}.

Paratypes: **4 ♀♀** mounted on card triangle, deposited at the LUNZ. Labels: ‘NEW ZEALAND, MC / Lincoln, Mahoe Reserve / 17 Jul 2015 F. Martoni / on *Acacia baileyana’* {Printed on white card}, ‘ID 180—Martoni F. 2017 / PhD Thesis. Lincoln University, / Canterbury–New Zealand.’ {Printed on white card}, ‘PARATYPE ♀ / *Acizzia errabunda* / Martoni & Armstrong 2019’ {Printed on blue card}; **2 ♀♀, 2 ♂♂** mounted on card triangle, deposited at the BMNH. Labels: ‘NEW ZEALAND, MC / Lincoln, Mahoe Reserve / 17 Jul 2015 F. Martoni / on *Acacia baileyana’* {Printed on white card}, ‘ID 180—Martoni F. 2017 / PhD Thesis. Lincoln University, / Canterbury–New Zealand.’ {Printed on white card}, ‘PARATYPE ♀ / *Acizzia errabunda* / Martoni & Armstrong 2019’ {Printed on blue card}; **2 ♀♀, 2 ♂♂** mounted on microscope slide, deposited at the LUNZ. Labels: ‘PARATYPE / *Acizzia errabunda* / Martoni & Armstrong / 2019’ {Hand-written on white card}, ‘AUSTRALIA, SA—Mt. Barker / 35°04’140”S 138°52’992”E / 6 Nov 2014—F. Martoni & G. Taylor / On *Acacia baileyana*’ {Hand-written on white card}.

### Diagnosis

*Acizzia errabunda* can be identified by the following combination of characters: intermediate body size, light colour of the body, including the antennae. Male proctiger elongated with apical part thin, parameres thin at the base, slightly back-turned, but with the tip facing the opposite direction and with a clear inner spike that can be easily seen from the lateral view. Female genitalia relatively short, with proctiger pronouncedly down-turned.

**Fig 7:**
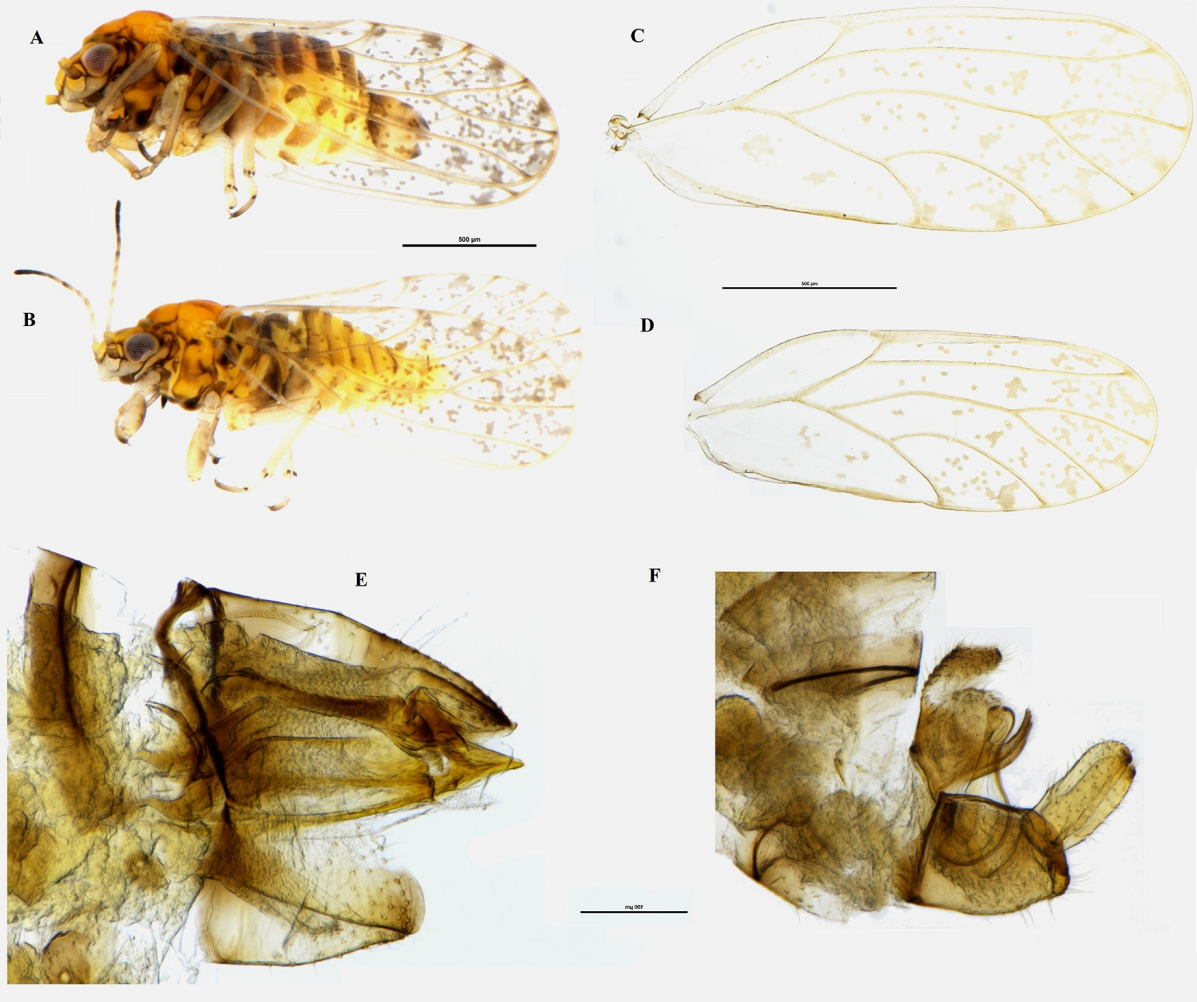
Acizzia acaciaebaileyanae. Habitus lateral view of female **(A)** and male **(B)**; wings of female **(C)** and male **(D)**; lateral view of terminalia of female **(E)** and male **(F)**. Scale bar length = 500μm **(A-D)** and 200μm **(E-F)**.

*Acizzia errabunda* can be easily distinguished from *Acizzia acaciaebaileyanae* (Froggatt, 1901) (Fig 7), the only other taxon inhabiting *Acacia baileyana*, by a number of characters. Firstly, *A*. *errabunda* shows a lighter colour of the body (Fig 5A–5D) compared to that of *A*. *acaciaebaileyanae* (Fig 7), more pronounced genal processes (especially in the females; Fig 5G and 5H) and the lack of a darker colour of the four terminal segments of the antennae as is the case in *A*. *acaciaebaileyanae* (Fig 7B). Furthermore, the male proctiger of *A*. *errabunda* is more elongated (Figs 5J, 3C and 3D), with the apical part thinner than that of *A*. *acaciaebaileyanae* (Fig 7F). The parameres of *A*. *errabunda* are thinner apically, slightly back-turned (but with the tip protruding in the opposite direction) and with an inner spike visible in lateral view that is absent in *A*. *acaciaebaileyanae* (Fig 7F).

**Fig 8:**
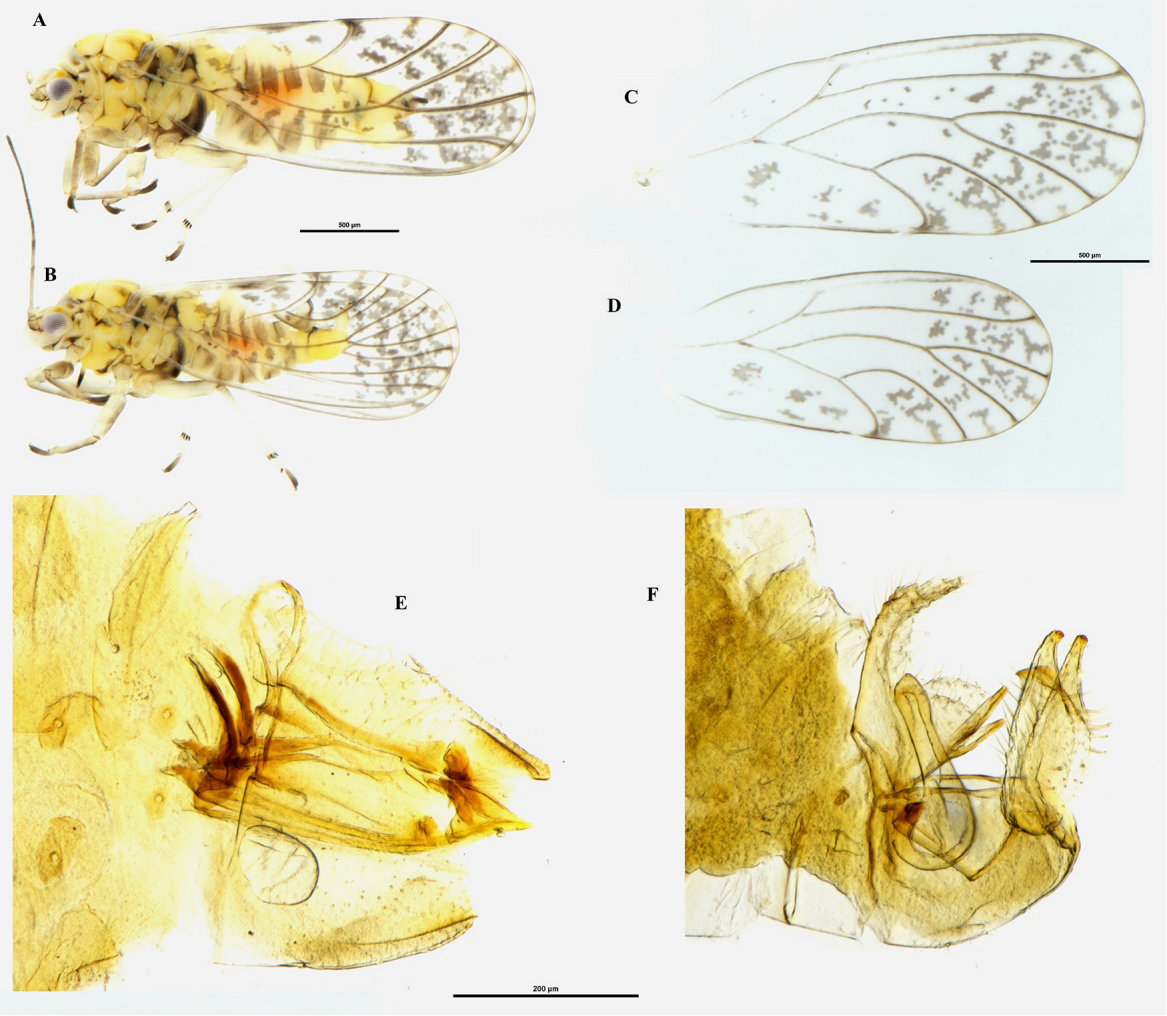
Acizzia jucunda. Habitus lateral view of female **(A)** and male **(B)**; wings of female **(C)** and male **(D)**; lateral view of terminalia of female **(E)** and male **(F)**. Scale bar length = 500μm **(A-D)** and 200μm **(E-F)**.

However, morphologically, *Acizzia errabunda* appears to be more similar to *Acizzia jucunda* (Fig 8), hosted by *Acacia decurrens* Willd. This is in agreement with the molecular results obtained through COI DNA barcoding that positioned these two species in closer genetic proximity to each other than any other species [18]. Both taxa have a similar male proctiger, with an apical part elongated and thin and a lower part shorter and wider. Yet *A*. *errabunda* presents a more curved hook that departs from the base of the proctiger shaping a double curve (Figs 5J, 3C and 3D), while that of *A*. *jucunda* appears to form a straighter line from the base of the proctiger and it is more clearly visible due to a darker colour (Fig 8F). Moreover, the parameres of *A*. *jucunda* are wider at the base compared to those of *A*. *errabunda*, and the distance between the inner spike and the tip of the parameres is more pronounced in *A*. *errabunda*. The female terminalia of *A*. *errabunda* have a proctiger with a pronounced down-turned tip (Figs 5I, 3A and 3B), while the terminalia of *A*. *jucunda* are less down-turned (Fig 8E).

#### Colouration

**Adult**. Body orange/brown, with head and pronotum orange or light orange, while the thorax is usually darker tending to brown (Fig 5A–5D). Pronotum with pale/white bands. Abdomen orange, with darker stripes in ventral position. Eyes dark brown. Genal processes light orange or yellow. Antennal segments uniformly light orange. Wings are transparent with darker veins and brown spots. The “Y” shaped spots formed by the radular spinules, often present in *Acizzia* wings, are here usually (but not always) incomplete (Fig 5E and 5F). Male terminalia are pale yellow with a black inner spike visible in lateral view. Female terminalia are slightly darker, with an orange proctiger, similar to the colour of the thorax, and a darker tip.

**Nymph**. Habitus as in Fig 6. The cephalo-thoracic plate is orange/yellow; eyes are brown; antennae light brown, with the last two segments almost black and the previous one of a darker brown. Thoracic sclerites brown; forewing and hindwing pads orange; caudal plate slightly glossy and of a darker orange.

#### Structure

**Adult**. Body quite short, with females about 1.5mm and males always shorter. Head: vertex quite diverse between males and females, with the latter showing a straight border between this and the thorax. Genal processes pronounced, especially in females, with the apical part ending in an irregular pointed tip. Antennae moderately long, at least 1.5 times as long as head.

Fore wing at least 3 times as long as width of head in males and almost 4 times in females; and about 2.5 times as long as wide. Veins M shorter than Rs, or rarely equal to it.

Male terminalia quite slender, with proctiger as long as parameres and thinner in apical part. Parameres thin and slender overall pointing backward but with the tip slightly turned forward. An internal spike pointing forward can also be seen in lateral view.

Female Terminalia straight and short with proctiger as long as subgenital plate or slightly longer. The terminal part of the proctiger ends in slightly darker and very lightly downturned tip.

**Nymph**. Setae are present on the head, on the legs and on the wings. Between 7 and 10 capitate setae on lateral margin of forewing pad and at least 5–7 capitate setae on apex of hindwing pad. On the lateral margin of the caudal plate are another 20–30 setae. The circum-anal pore ring is located in ventral position.

#### Measurements and ratios

Measurements are in mm (5 ♂, 5 ♀). Length of body (vertex to terminalia) ♂ 1.14–1.36, ♀ 1.52–1.64; length of body (vertex to apex of folded wings) ♂ 1.55–1.66, ♀ 1.83–2.13; width of head (HW) ♂ 0.40–0.42, ♀ 0.45–0.48; length of genal processes (GCL) ♂ 0.04–0.05, ♀ 0.07–0.09; length of vertex (VL) ♂ 0.12–0.13, ♀ 0.16–0.19; width of vertex (VW) ♂ 0.25–0.26, ♀ 0.26–0.30; length of antenna (AL) ♂ 0.66–0.69, ♀ 0.72–0.77; length of fore wing ♂ 1.22–1.42, ♀ 1.62–1.91; width of fore wing ♂ 0.45–0.59, ♀ 0.68–0.77; length of vein Rs ♂ 0.67–0.81, ♀ 1.02–1.21; length of vein M (M) ♂ 0.41–0.51, ♀ 0.56–0.71; length of vein M1+2 (M1) ♂ 0.36–0.43, ♀ 0.56–0.71; marginal width of cell m1 ♂ 0.15–0.19, ♀ 0.22–0.27; marginal width of cell cu1 ♂ 0.25–0.30, ♀ 0.35–0.47; length of vein Cu1b ♂ 0.15–0.22, ♀ 0.22–0.29; length (height) of proctiger (PL) ♂ 0.20–0.21; length of paramere ♂ 0.19–0.20; length of proximal aedeagal segment ♂ 0.28–0.29; length of distal aedeagal segment ♂ 0.14–0.15; length of proctiger (PL) ♀ 0.26–0.31; length of circum-anal ring (CL) ♀ 0.13–0.14; length of subgenital plate (SL) ♀ 0.26–0.27.

Ratios: GCL:VL ♂ 0.36–0.42, ♀ 0.38–0.52; VL:VW ♂ 0.47–0.49, ♀ 0.56–0.71; VL:HW ♂ 0.29–0.30, ♀ 0.34–0.41; AL:HW ♂ 1.59–1.70, ♀ 1.55–1.62; PL:HW ♂ 0.47–0.53, ♀ 0.61–0.65; PL:CL ♀ 1.88–2.28; PL:SL ♀ 1.08–1.25; WL:HW ♂ 3.05–3.58, ♀ 3.60–3.94; WL:WW ♂ 2.41–2.70, ♀ 2.36–2.56; Rs:M ♂ 1.58–1.72, ♀ 1.71–1.99; M1:M ♂ 0.83–0.97, ♀ 0.94–1.26.

#### Remarks

*Acizzia errabunda* was not reported in the most recent checklist of the New Zealand psyllids [22], but was collected more recently around Auckland by S. Thorpe and uploaded as observation on the iNaturalistNZ website as “*Acizzia* st2” [44].

### Etymology

The word “errabunda” means “wanderer” in Latin. This name was chosen to indicate the fact *A*. *errabunda* travelled between Australia and New Zealand before being described.

#### Distribution

*Acizzia errabunda* was recorded in two populations in New Zealand and one in Australia (Table 1). The New Zealand populations were collected in the Mahoe reserve, in the town of Lincoln, and in Spring Grove, near Nelson. The Australian population was collected in Mt. Barker, South Australia. Furthermore, the distribution of *A*. *errabunda* reported here, is also inferred by pictures taken of individuals collected from three population in the urban area of Auckland (Table 1; S. Thorpe, [44]).

#### Host plant

All the psyllid populations examined were collected from *Acacia baileyana* (Fabaceae) and presented a high number of individuals. The finding of immature stages on the plant allows us to report *A*. *baileyana* as the host plant. This is also known as the Cootamundra wattle, and it is indigenous to New South Wales, Australia, but has been extensively introduced to New Zealand (both North and South Island), as well as other countries such as Europe, South America and South Africa [45].

## Discussion

The morphology of the psyllids analysed here parallels the extent of DNA barcode divergence results for these two taxa and justifies their description as new species. In the case of *Acizzia errabunda*, the morphology also confirmed a strong similarity with the species *A*. *jucunda*, which was reported to be the most closely related species based on COI divergence [18]. With the data presented here, the New Zealand psyllid fauna is now composed of 73 described species, divided into six families and 24 genera, and more undescribed taxa ranging between 27 and 47 [18, 22, 26]. The presence of *A*. *errabunda* and *C*. *insularis* in New Zealand and their association with non-native host plants suggests that these species are amongst the 36 psyllid taxa adventive to the archipelago. Their arrival to New Zealand is hypothesised here to be contemporaneous to that of the host plants, especially considering both plant species are ornamental and widely planted in private and public gardens [44]. This suggests that constant monitoring of the New Zealand biodiversity is key to a prompt record of adventive taxa. In fact, both the species described here had been previously observed and reported on the iNaturalist online database together with pictures highlighting their morphological characters. This allowed a morphological examination that resulted in the identification of the taxa and, consequently, the distribution of these psyllids was strongly improved by the observations available. This confirms the importance of “citizen science” [46] in enabling a better understanding of the distribution and diversity of insects even in urban areas; and supports a number of previous studies (e.g. [47–49]). Similarly, the availability of museum specimens allowed additional insights in the distribution of these two new species, highlighting the fundamental importance of entomological collections and museums in the understanding of worldwide biodiversity. Indeed, the description of the two new species reported here enables the number of species belonging to their respective genera to be updated, in New Zealand and worldwide.

The origin of *Ctenarytaina insularis* still requires elucidation, since *Syzygium smithii* is not native to New Zealand and *Melaleuca* is not present here, this psyllid is probably not native to this country. Based on host plant association *C*. *insularis* may be native to New Caledonia where it is hosted by a native plant, *Melaleuca quinquenervia*. However, this host is native not only to New Caledonia, but also to Papua New Guinea and the Eastern coast of Australia [50], suggesting *C*. *insularis* could be native to any one of these areas. While no record of this psyllid species has been reported from Australia or Papua New Guinea, future collection may confirm its presence in these countries. The host switch to *Syzygium* may have happened in Brunei or in the nearby areas of Malaysia and Indonesia, where also *Melaleuca* is present [51], although *C*. *insularis* has not yet been recorded there either. Therefore, until further collections are performed, the most plausible origin of *C*. *insularis* appears to be New Caledonia. This is also confirmed by its wide distribution on this island, and by the number of different locations where it was collected in 1982. Hopefully, the present record of this species can improve the understanding not only of New Zealand’s biodiversity, but also that of Australia, Brunei and New Caledonia, as well as stimulate further study of their respective psyllid faunas.

When studying the Australian *Acizzia*, in 1980, Yen reported the presence of at least three morphospecies on *Acacia baileyana*: *A*. *acaciaebaileyanae* and two undescribed taxa [52]. This suggests *A*. *errabunda* was probably included in Yen’s work as one of the two undescribed morphospecies [52]. Ultimately, the origin of *A*. *errabunda* appears to be Australian based both on its presence there and on the origin of its host plant.

In New Zealand, with the addition of *A*. *errabunda*, the genus *Acizzia* is now represented by 11 described species [22].
